# Measurement of the Spatial Distribution of S1P in Small Quantities of Tissues: Development and Application of a Highly Sensitive LC-MS/MS Method Combined with Laser Microdissection

**DOI:** 10.5702/massspectrometry.A0072

**Published:** 2019-02-14

**Authors:** Jiao Wang, Kuniyuki Kano, Daisuke Saigusa, Junken Aoki

**Affiliations:** 1 Laboratory of Molecular and Cellular Biochemistry, Graduate School of Pharmaceutical Sciences, Tohoku University; 2 Department of Integrative Genomics, Tohoku Medical Megabank Organization, Tohoku University; 3 Medical Biochemistry, Tohoku University School of Medicine; 4 AMED·LEAP

**Keywords:** sphingosine-1-phosphate, laser microdissection, column-switching LC-MS/MS

## Abstract

Sphingosine-1-phosphate (S1P) acts as an extracellular signaling molecule with diverse biological functions. Tissues appear to have an S1P gradient, which is functionally relevant in the biological significance of S1P, although its existence has not been measured directly. Here, we report a highly sensitive method to determine the distribution of S1P, using a column-switching LC-MS/MS system combined with laser microdissection (LMD). Column switching using narrow core Capcell Pak C18 analytical and trap columns with 0.3 mm inner diameter improved the performance of the LC-MS/MS system. The calibration curve of S1P showed good linearity (*r*>0.999) over the range of 0.05–10 nM (1–200 fmol/injection). The accuracy of the method was confirmed by measuring S1P-spiked laser microdissected mice tissue sections. To evaluate our S1P analytical method, we quantified S1P extracted from micro-dissected mouse brain and spleen. These results show that this method can measure low S1P concentrations and determine S1P distribution in tissue microenvironments.

## INTRODUCTION

Sphingosine-1-phosphate (S1P), an important cell-signaling molecule, mediates various pathophysiological processes, such as autoimmunity and vascular development, *via* five cell surface receptors (S1P_1–5_).^1,2)^ S1P is intracellularly synthesized by the phosphorylation of sphingosine in multiple cells,^3)^ and then exported to the extracellular environment by specific transporters.^4)^ S1P concentration is much higher in blood and lymphatic fluid than in interstitial spaces in lymph organs due to the activity of S1P lyase. This S1P gradient causes lymphocytes to move from lymph organs to the circulation through S1P_1_ receptor expressed on lymphocyte.^5–7)^ In addition, mapping S1P in mice by analyzing S1P receptor signaling suggested the presence of a local S1P gradient within tissues.^8–11)^ For example, S1P concentration seems to decrease with distance from the marginal zone in the white pulp.^10)^ However, the existence of an S1P gradient within tissue has not yet been directly measured.

Lipid analysis has been improved greatly in recent years through advances in liquid chromatography (LC) coupled with MS/MS systems. Previously, the limit of detection for sphingolipids had been reduced to the nanomolar level.^12)^ Although LC-MS/MS has been used widely in lipid research due to its ability to quantify lipids, it is not well-suited for analyzing tissue samples because it requires sample preparation, which destroys spatial information about the analyte. To overcome this problem, imaging mass spectrometry, such as matrix assisted laser desorption ionization-imaging mass spectrometry, has been developed to visualize the spatial distribution of analytes in tissue samples.^13)^ This technique can clearly visualize sphingolipids including sphingomyelin (SM), which is abundant lipid in eukaryotic cells, in tissue sections. However, imaging S1P with high spatial resolution is challenging because its level is much lower than that of SM. Therefore, we sought to develop a more sensitive method to determine the level of S1P with spatial information.

Laser microdissection (LMD) is a contact- and contamination-free method for isolating specific single cells or small areas from a wide variety of samples. LMD is now used in a large number of research fields, such as proteomics, cancer research, neuroscience, and plant research.^14)^ For instance, the combination of LMD with LC-MS has been successfully applied to explore protein distribution pattern.^15)^ Previously, we utilized LC-MS/MS combined LMD to quantify sphingolipids in small liver samples.^16)^ To further improve the performance for S1P, we combined the column-switching LC-MS/MS method with a narrow core column. Column-switching is a two-column liquid chromatography technique that has been used for chromatographic separation, on-line sample cleanup, deproteinization, and trace enrichment.^17)^ To our knowledge, our method is the highest sensitive one that coupled LMD with LC-MS/MS to investigate lipid tissue distribution.

## MATERIALS AND METHODS

### Reagents

C18-sphingosine-1-phosphate (C18-S1P) and C17-dihydrosphingosine-1-phosphate (C17-dhS1P) were purchased from Avanti Polar Lipids Inc. (Alabaster, AL). All chemicals and solvents were of analytical grade.

### Chromatographic conditions

A Dionex UltiMate 3000 (Thermo Scientific) LC system was used. Three reverse phase columns with different inner diameters and length were utilized for LC optimization, including Capcell Pak C18 (150 mm×1.5 mm I.D., 3 μm particle size), Capcell Pak C18 (150 mm×0.3 mm I.D., 3 μm particle size) and Capcell Pak C18 (50 mm×0.3 mm I.D., 3 μm particle size). Capcell Pak C18 (150 mm×0.3 mm I.D., 3 μm particle size) was applied as analytical column and Capcell Pak C18 (50 mm×0.3 mm I.D., 3 μm particle size) as trap column for column switching system. Columns were maintained at 40°C. The mobile phase was 5 mmol/L ammonium formate (HCOONH_4_)–H_2_O (pH 4) (A) and 5 mmol/L HCOONH_4_–H_2_O/acetonitrile (CH_3_CN) (5 : 95, v/v; pH 4) (B), which was detailed in an earlier study.^18)^ For the analysis with 150 mm×1.5 mm-I.D. column, the elution gradient with flow rate of 200 μL/min started with 50% B for 0.2 min, increasing to 100% B in 2.7 min, held for 2.9 min and then back to initial conditions. The column was re-equilibrated for 1.1 min until the next injection. For the analysis with 150 mm×0.3 mm-I.D. column, the elution gradient the elution gradient with flow rate of 20 μL/min started with 30% B for 1 min, increasing to 85% B in 5 min and then to 100% in 4 min. After held in 100% B for 10 min, gradient went back to initial conditions and column was re-equilibrated for 15 min until another injection. The gradient program for column switching system was as follows: the elution with the flow rate of 10 μL/min from first pump for the separation column was first 30% B for 8 min, the proportion of B then was linearly increased to 100% B in 7 min, it was held for 15 min and immediately returned to the initial condition and maintained for another 15 min until the end of the run, the elution from the second pump for the trap column was isocratic, with 30% B of 10 μL/min, valve was switched at 10 min to change the direction of elution.

### Mass spectrometric conditions

The MS system was a TSQ Quantiva (Thermo Fisher Scientific, San Jose, CA) triple quadrupole mass spectrometer equipped with a heated-electrospray ionization-II (HESI-II) source. The nebulizing gas and collision gas were nitrogen and argon, respectively. Standard solutions dissolved in MeOH (1.0 μmol/L) were infused into MS continuously by a syringe pump at the rate of 5 μL/min. HESI was performed in positive mode for S1P. Samples were analyzed in SRM mode, using the transitions of the [M+H]^+^ precursor ions to their product ions. The MS/MS transitions were determined in full scan mode (*m*/*z* 50–450). Optimized parameters are described in Table 1.

**Table 1:** Table 1. Optimal conditions for MS analysis of sphingosine-1-phosphate.

MS system	TSQ Quantiva (Thermo Fisher Scientific)
Ionization	ESI(+)
Spray voltage	3.0 kV
Vaporizer temperature	350°C
Sheath gas pressure	65 psi
Auxiliary gas pressure	20 psi
Capillary temperature	350°C
Collision gas pressure	1.5 mTorr
RF lens offset	C17 dhS1P: 86 V
	C18 S1P: 86 V
Collision energy	C17 dhS1P: 16 eV (*m*/*z* 368.3→270.2)
	C18 S1P: 18 eV (*m*/*z* 380.3→264.2)

### Validation parameters

A 10 μmol/L stock solution of S1P was prepared in methanol. It was evaporated and resuspended in 0.1% BSA-PBS and diluted to the concentration of 0.125, 0.25, 1.25, 2.5, 12.5, 25 μmol/L as standard solution. C17-dhS1P (100 nmol/L, final concentration) was selected as the internal standard (IS) in the present method. We confirmed that ion intensity and peak sharpness of C17-dhS1P were almost equal to those of C17-S1P, which is another commercially available IS. Standard calibration solutions were prepared with LMD-dissected tissue fragments (liver, spleen, kidney, brain) obtained from male C57BL/6 mice. 0.2 μL of each standard solution were spiked on the surface of tissue cryosections and dried in room temperature. LMD-dissected sections were collected with 500 μL IS solution added in each tube. The obtained mixture was homogenized for 10 min in an ultrasonic bath. After centrifugation at 15,000×g for 10 min at 4°C, the supernatant was collected, filtered and centrifuged at 6,500×g for 5 min at 4°C. An aliquot (20 μL) of filtered solution was analyzed by column-switching LC-MS/MS. For validation of the method, samples prepared at concentrations of 1, 2, 10, 20, 100 and 200 fmol/injection were injected four times on the same day and analyzed. This procedure was repeated for 3 times in independent experiments. The accuracy was calculated as [(found concentration−endogenous concentration)/spiked concentration−1]×100 (%), and the precision was evaluated using the coefficient of variation. Peak areas and calibration curves were obtained using Xcalibur software (Thermo Fisher Scientific).

### Preparation of biological samples

Mice tissues (liver, spleen, kidney, brain) were obtained from male C57BL/6 mice. Mice were first anesthetized with ethyl carbamate and then rapidly perfused transcardially with PBS through the left ventricle and sacrificed. Tissue was quickly removed, embedded in OCT compound and snap frozen by liquid nitrogen. Cryosections of 15 μm thickness were made with a Leica CM1950 cryostat. Tissue sections attached to PEN membrane glass slides were laser microdissected (Leica LMD7000). To dissect spleen white pulp and red pulp, more than 20 spleen slices stained by 0.5% toluidine blue were microdissected and the tissue dissectates of the same sub-region were pooled. We confirmed that the level of S1P in spleen section was unchanged by staining. Laser parameters for dissection were laser power: 45, aperture: 20 and speed: 10. Five hundred μL IS (100 nmol/L) was added to LMD-dissected sections. S1P was extracted from the dissected samples as described in the ‘Method validation’ section.

## RESULTS AND DISCUSSION

### LC-MS/MS method development

**Figure 1:**
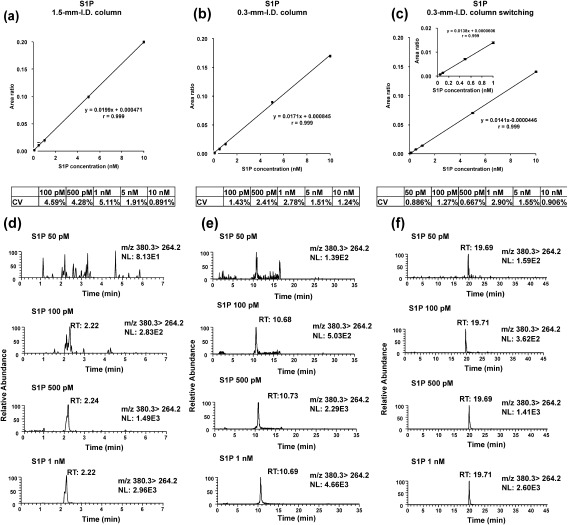
Fig. 1. Performance of optimized LC-MS/MS systems for S1P quantification.

In order to improve LC-MS/MS detection of S1P, a highly sensitive mass spectrometer, TSQ Quantiva (Thermo Scientific) was used. The optimized parameters are shown in Table 1. SRM transitions are *m*/*z* 368.3→270.2 for C17-dhS1P and 380.3→264.2 for C18-S1P. The quantification limit of S1P (signal to noise ratio of ≥10) was 100 pM (2 fmol/injection) (Fig. 1a, d). To improve peak sharpness, we used a narrow (0.3 mm I.D.) C18 column with a low elution flow rate. We obtained a symmetrical peak shape for S1P using the narrow column. Although the narrow column and low flow rate did not improve the detection limit, the precision, as expressed by the coefficient of variation (CV), was improved (Fig. 1b, e). We then added column switching, which is a technique for concentrating an analyte. In these systems, the analyte is first trapped in a trap column and then the direction of elution is switched to back-flush the enriched analyte to an analytical column. With this system, the detection limit for S1P fell to 50 pM (1 fmol/injection) (Fig. 1c, f), a 20-fold improvement compared with a previously reported method.^9)^

(a–c) Standard curves and CV% of S1P quantified by TSQ Quantiva with 1.5-mm-I.D. column, 0.3-mm-I.D. column and 0.3-mm-I.D. column switching systems. (d–f) Chromatograms of S1P measured by the same methods of (a–c).

### Method validation

**Figure 2:**
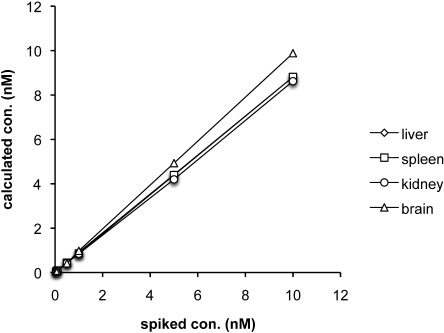
Fig. 2. Comparison of the calculated and spiked S1P concentrations in different tissues.

To evaluate the method, we examined linearity, recovery, precision and the study of matrix effects. The relative standard deviation ranged from 0.429 to 3.06% for the 0.125 μmol/L spiked samples and the recovery varied from 83.1 (–16.9) to 96.94 (−3.06)% (Table 2). Calibration curves for S1P were linear from 1 to 200 fmol/injection, fitted to a linear equation of the slope and intercept (*y*=*ax*+*b*). Correlation coefficients were better than 0.99 (Table 3). As shown in Fig. 2, the calculated S1P concentration that subtracted naturally presented S1P in naïve samples, against the spiked S1P concentration was plotted. Linear recoveries were yielded for each tissue samples. These data suggest that this method is accurate and reproducible for analyzing S1P in biological samples and demonstrate its reliability.

**Table 2:** Table 2. Accuracy and precision of determination method for sphingosine-1-phosphate.

	Intraday (*n*=4)						Interday (*n*=3)					
	Range (fmol/injection)						Range (fmol/injection)					
	1	2	10	20	100	200	1	2	10	20	100	200
Accuracy (%)												
Liver	−15.8	−15.2	−12.3	−8.47	−11.8	−11.9	−16.9	−13.1	−12.3	−12.7	−12.4	−12.1
Spleen	−5.81	−7.51	−10.7	−13.7	−12.2	−11.9	−3.06	−4.15	−9.82	−11.7	−11.2	−10.8
Kidney	−5.89	−8.81	−12.9	−15.3	−16.1	−13.8	−4.3	−8.95	−11.8	−14.9	−14.7	−13.8
Brain	−3.07	−5.61	−0.422	−6.88	−1.03	−0.105	−6.01	−9.24	−8.29	−2.65	−1.12	−0.608
Precision (%)												
Liver	1.21	0.743	1.79	2.4	0.747	0.399	3.06	5.43	1.86	4.91	2.13	2.43
Spleen	2.75	2.11	1.75	5.02	1.91	1.71	1.02	1.2	1.49	2.2	1.57	2.01
Kidney	1.1	1.76	1.57	1.16	3.58	2.17	1.08	0.708	1.72	2.03	1.35	0.45
Brain	1.92	2.74	4.58	2.43	1.38	1.16	0.429	0.0591	1.17	1.2	0.0725	0.378

**Table 3:** Table 3. S1P calibration curves.

Tissue	Equation^1^	*r*
Liver	*y*=0.881*x*+6.20	0.999
Spleen	*y*=0.881*x*−2.23	0.999
Kidney	*y*=0.859*x*−10.3	0.999
Brain	*y*=0.991*x*−23.9	0.999

^1^*y*=calculated concentration; *x*=spiked concentration.

S1P standard spiked on each LMD-dissected liver, spleen, kidney and brain tissue sections were extracted and applied to column-switching LC-MS/MS system.

### Application of LMD-combined column-switching LC-MS/MS for investigating distribution of S1P in brain and spleen tissue sections

**Figure 3:**
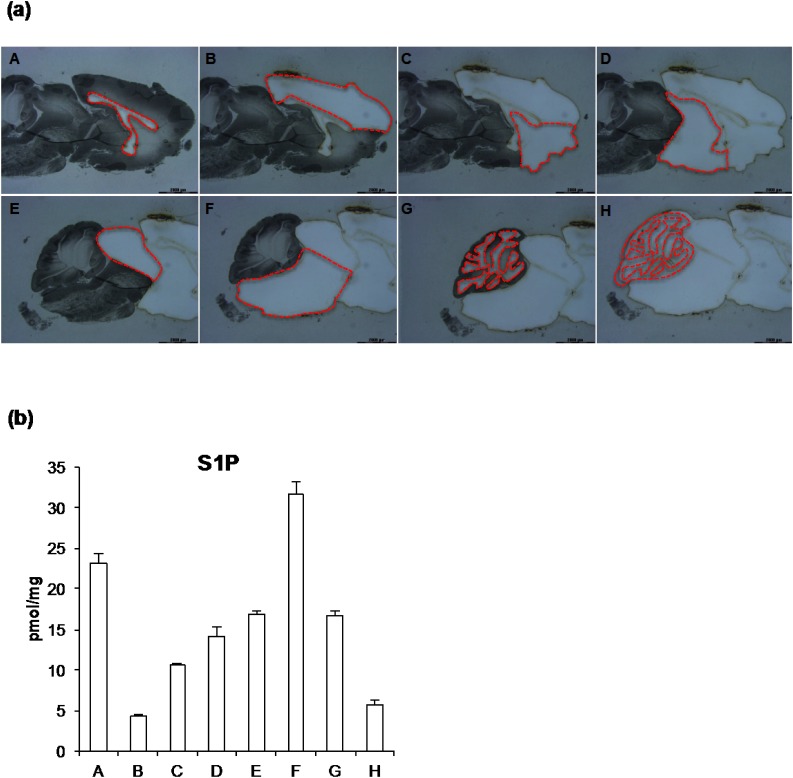
Fig. 3. S1P distribution in brain tissue sections.

**Figure 4:**
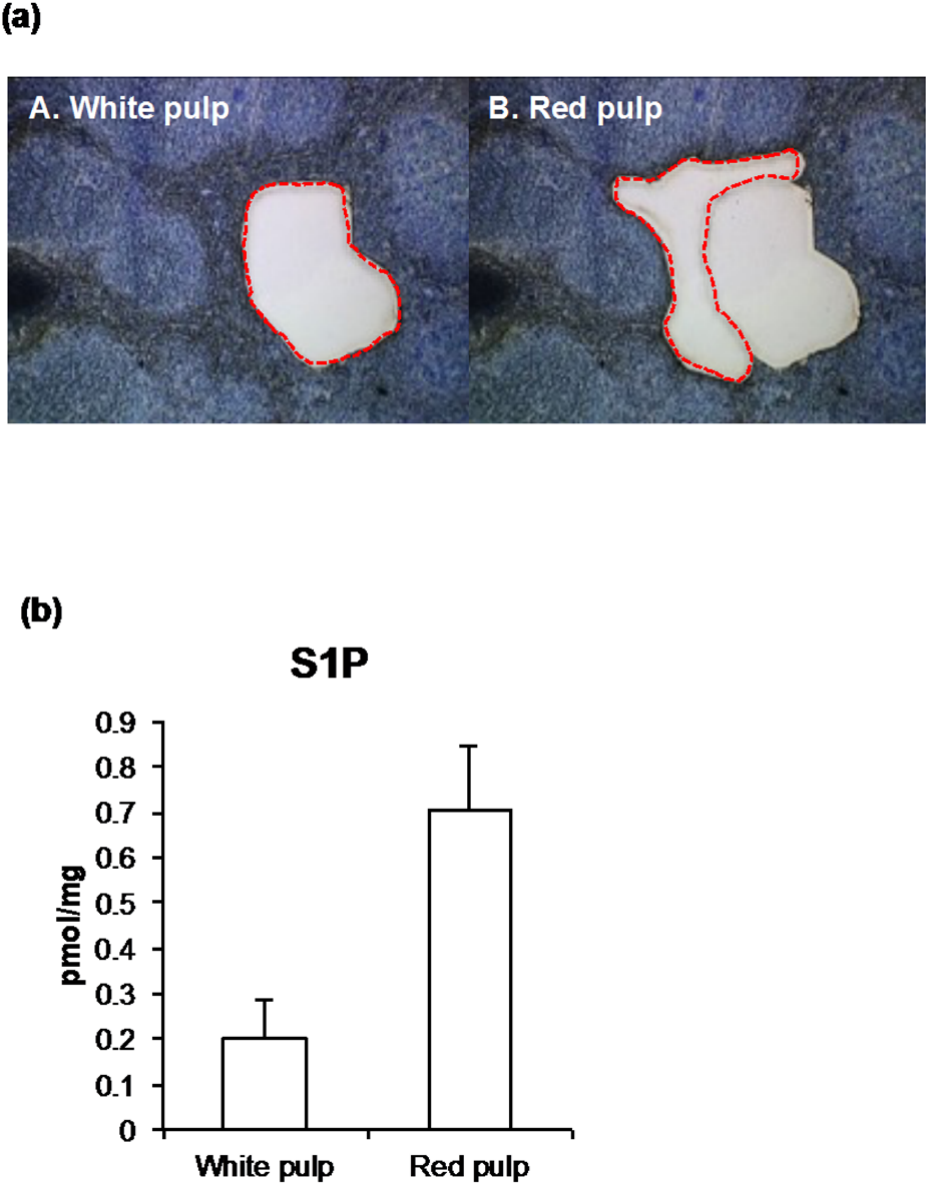
Fig. 4. S1P distribution in spleen tissue sections.

To test the utility of this technique, we examined the levels of S1P in different sub-regions of the brain and spleen. Brain is an S1P-rich tissue.^12)^ Furthermore, S1P-related molecules (sphingosine kinases, transporters and receptors) have different functions in different parts of the central nervous system.^19–21)^ To see these differences more clearly, we prepared eight transverse sections of an adult mouse brain and then microdissected parts of these sections corresponding to different brain parts (corpus callosum, cerebral cortex, *etc.*) (Fig. 3a). In this figure, the microdissected parts are indicated by dashed red lines. We then measured the level of S1P in each part. S1P was detected in each part, with the highest levels in the corpus callosum (Fig. 3b A), pons and medullar (Fig. 3b F). In the cerebellum, the level of S1P was significantly higher in the white matter (Fig. 3b G) than in the grey matter (Fig. 3b H). The cerebral cortex, which is the outer covering of the gray matter, also had a low level of S1P (Fig. 3b B). Unexpectedly, these differences in S1P levels were not consistent with the sphingosine kinase (SPHK) activity.^22)^ That is, high SPHK activity was found in cortex, which showed the lowest levels of S1P in the present study (Fig. 3b B). This discrepancy may be due to differences in the level of sphingosine that act as a substrate of SPHK, and in the S1P degradation by S1P lyase.^23,24)^ We also examined the distribution of S1P in the spleen. The spleen mainly contains two different tissues, red pulp and white pulp. Red pulp is connective tissue that receives circulating blood containing abundant S1P and removes abnormal red blood cells. Red pulp is composed of splenic cords and venous sinuses. Splenic cords are consisted from reticular fibers, myofibroblasts and macrophages, while venous sinuses are lined by endothelial cells. In contrast, white pulp is lymphatic tissue consisting of lymphocytes, macrophages, dendritic cells, plasma cells, arterioles, and capillaries in a reticular framework similar to that found in the red pulp.^25)^ In a spleen section, we microdissected a white pulp area and the adjacent red pulp area (Fig. 4a), and determined S1P level. The S1P concentrations in the spleen (<1 pmol/mg) were much lower than those in the brain (<35 pmol/mg). Interestingly, S1P concentration was higher in red pulp than in white pulp (Fig. 4b). However, it was previously reported that mouse red pulp had little signaling-available S1P,^10)^ the distribution of S1P remains controversial.

Frozen 15-μm brain sections were dissected by LMD. S1P was extracted from the dissectates (see Methods) and analyzed by column-switching LC-MS/MS. (a) Figures of dissected brain tissue sections by LMD. The microdissected regions are indicated by dashed red lines. A: corpus callosum, B: cerebral cortex, C: anterior olfactory nucleus and striatum, D: hippocampus, thalamus and hypothalamus, E: superior colliculus, inferior colliculus and midbrain reticular nucleus F: pons and medullar, G: white matter of cerebellum, H: Grey matter of cerebellum. (b) Concentration of S1P in distinct LMD-dissected brain sub-regions.

More than 20 splices of frozen 15-μm, 0.5% toluidine blue stained spleen sections were dissected by LMD. S1P was extracted from the pooled dissectates of the same sub-region (white pulp or red pulp) (see Methods) and analyzed by column-switching LC-MS/MS. (a) Figures of dissected spleen tissue sections by LMD. The microdissected regions are indicated by dashed red lines. (b) Concentration of S1P in distinct LMD-dissected spleen sub-regions.

## CONCLUSION

Our LC-MS/MS system, which couples a 0.3-mm-I.D. column-switching technique to a TSQ Quantiva™ mass spectrometer, increased the sensitivity to S1P dramatically, reaching a detection limit of 50 pM (1 fmol/injection). The system also had good linearity, recoveries and precision. Our analyses of mouse brain and spleen samples demonstrate that combining column-switching LC-MS/MS with laser microdissection is feasible for studying the distribution of S1P in tissue sections. The technique developed in this study will allow further analysis for other lipids in the future.

## Abbreviations

S1P, sphingosine-1-phosphate; LMD, laser microdissection; LC, liquid chromatography; MS, mass spectrometry; MALDI-IMS, matrix assisted laser desorption/ionization-imaging mass spectrometry; SRM, selected reaction monitoring; IS, internal standard

## Notes

Mass Spectrom (Tokyo) 2019; 8(1): A0072
